# A helping hand: roles for accessory cells in the sense of touch across species

**DOI:** 10.3389/fncel.2024.1367476

**Published:** 2024-02-16

**Authors:** David R. Logan, Jesse Hall, Laura Bianchi

**Affiliations:** Department of Physiology and Biophysics, University of Miami, Miami, FL, United States

**Keywords:** touch, mechanotransduction, accessory cells, glia, *C. elegans*, *Drosophila*

## Abstract

During touch, mechanical forces are converted into electrochemical signals by tactile organs made of neurons, accessory cells, and their shared extracellular spaces. Accessory cells, including Merkel cells, keratinocytes, lamellar cells, and glia, play an important role in the sensation of touch. In some cases, these cells are intrinsically mechanosensitive; however, other roles include the release of chemical messengers, the chemical modification of spaces that are shared with neurons, and the tuning of neural sensitivity by direct physical contact. Despite great progress in the last decade, the precise roles of these cells in the sense of touch remains unclear. Here we review the known and hypothesized contributions of several accessory cells to touch by incorporating research from multiple organisms including *C. elegans*, *D. melanogaster*, mammals, avian models, and plants. Several broad parallels are identified including the regulation of extracellular ions and the release of neuromodulators by accessory cells, as well as the emerging potential physical contact between accessory cells and sensory neurons via tethers. Our broader perspective incorporates the importance of accessory cells to the understanding of human touch and pain, as well as to animal touch and its molecular underpinnings, which are underrepresented among the animal welfare literature. A greater understanding of touch, which must include a role for accessory cells, is also relevant to emergent technical applications including prosthetics, virtual reality, and robotics.

## Introduction

In many organisms, mechanoreception is mediated by organs composed of at least two different cell types: neurons and accessory cells, including glia. This review concerns the contribution of accessory cells to touch, many of which are intrinsically mechanosensitive. These cells have historically been underappreciated for their active role in the sense of touch. For example, the role of keratinocytes as intrinsic mechanoreceptors and as contributors to mammalian touch and pain has been identified mostly in the last decade (Baumbauer et al., 2015; Moehring et al., 2018; Mikesell et al., 2022). A greater discernment of the roles for accessory cells in mechanoreception is important to understand disease processes. For example, maladies like hyperalgesia, a condition in which there is exaggerated pain, and allodynia, a condition in which innocuous stimuli cause pain, affect up to half of all patients with neuropathic pain (Jensen and Finnerup, 2014). In these maladies, pain is produced by innocuous touch and seems to, at least in part, depend on the accessory cells of touch receptors. Indeed, Merkel cells, a type of epidermal accessory cell reviewed in detail below, are thought to participate in mechanical allodynia, mechanical itch, and mechanical alloknesis, an itch sensation evoked by mechanical stimuli that normally do not evoke itch (Zhang et al., 2002; Bataille-Savattier et al., 2023). Furthermore, serotonin released by the Merkel cells may play a role in paresthesia, abnormal sensations such as tingling or prickling, associated with the use or withdrawal of popular serotonin uptake inhibitors (Praharaj, 2004; Chang and Gu, 2020a). Finally, a previously uncharacterized sensory organ of specialized glia was found important for painful touch in mice (Abdo et al., 2019).

Progress in the basic mechanisms for mechanoreception may also be translatable to industrial applications for which the sense of touch is needed, but for which the mechanoreceptors and their appendant molecular machinery are not present. For example, a greater understanding of mechanoreception is essential for prosthetics to be felt as a “greater part of one’s body” (Bartolozzi et al., 2016; Wang et al., 2021). In addition, since the advent of virtual reality (VR), a further understanding of the basic science of touch is needed to reproduce the perception of being in another location. However, at present, adding touch to visual-only interfaces remains a crucial impediment to the VR industry (See et al., 2022).

Expanding the study of touch to include other organisms is helpful to reveal the basic principles of touch. For example, the presence of lamellar touch corpuscles in a variety of species has helped to identify broad structure-function relationships via physical modeling (Quindlen-Hotek et al., 2020). In some cases, such as ducks or zebrafish, cells and organs which are important to the study of touch develop in a manner that is amenable to experimental design (Nikolaev et al., 2020; Brown et al., 2023). Furthermore, organisms such as *C. elegans* and *D. melanogaster* offer anatomical simplicity, genetic amenability, and other technical advantages that can accelerate discoveries into the cellular and molecular mechanisms underlying the interaction between accessory cells and sensory neurons in touch (Han et al., 2013; Singhvi and Shaham, 2019; Johnson et al., 2020; Wang and Bianchi, 2020; Prelic et al., 2021; Fernandez-Abascal et al., 2022; Mangione et al., 2023). The model emerging so far from studies across species is one in which cooperation and coordination between sensory endings and accessory cells mediate response to a range of mechanical forces that contribute to the experience of touch sensation.

## Merkel cells

**FIGURE 1:**
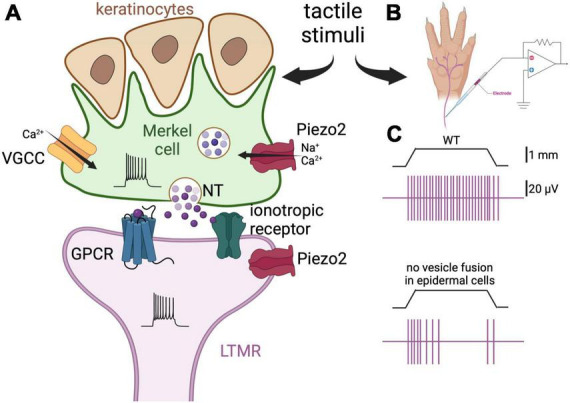
Merkel cells are touch-sensing and touch-transducing cells. **(A)** Depiction of a Merkel cell (green) and LTMR (purple) complex. Merkel cells are epidermal cells that form synapses with slowly adapting LTMR and are responsible for the detection of light touch. They are found in abundance in fingers and around the lips, and are associated with hair follicles. Merkel cells respond to mechanical stimulation by activation of the mechanosensitive cationic channel Piezo2 (Ikeda et al., 2014; Ranade et al., 2014; Woo et al., 2014). Consequently, membrane depolarization and Ca^2+^ influx induce neurotransmitter (NT) release by way of SNARE-mediated vesicle release (Hoffman et al., 2018). There is controversy about which neurotransmitter is released by Merkel cells, with serotonin, glutamate, and adrenaline all being implicated (Chang et al., 2016, 2017; Hoffman et al., 2018; Higashikawa et al., 2019; Sonekatsu et al., 2019; Chang and Gu, 2020a). Finally, the neurotransmitter interacts with G-protein-coupled receptors (GPCR) or ionotropic receptors on the afferent fibers leading to increased firing of action potentials. The postulated receptors for serotonin, glutamate, and adrenaline are the 5-HT3 receptor, the NMDA and glutamate-permeable anion channels, and the β2 adrenergic receptor, respectively (Chang et al., 2016, 2017; Hoffman et al., 2018; Higashikawa et al., 2019). Piezo2 channels are also expressed on the afferents, thus mediating concomitant mechanical activation of the nerve fibers. Merkel cells also form finger-like projections apically, where they interact with keratinocytes. **(B)** Schematic representation of the skin-nerve preparation used to record the electrophysiological activity of the nerve endings of the mouse skin upon touch stimulation. Normally, the hindlimb skin and saphenous of the mouse are used and mechanical stimulation is via von Frey monofilaments. **(C)** Top panel, depiction of a typical response of a LTMR to 1 mm indentation. The vertical purple bars represent action potentials. Bottom panel, the same stimulation elicits fewer action potentials in a fiber of a mouse in which the tetanus neuro-toxin light-chain subunit was expressed in the Merkel cells, thus preventing SNARE-dependent vesicular fusion [adapted from Hoffman et al. (2018)]. These results support the idea that Merkel cells regulate LTMRs via release of neurotransmitter.

Merkel cells are epidermal cells with elliptical morphology that are characterized by electron-dense core granules, a paucity of cytoskeletal filaments, and indented nuclei (Merkel, 1875; Abraham and Mathew, 2019). Together with sensory afferents and columnar epithelia, Merkel cells make up part of the widespread epidermal structure known as the touch dome (Lumpkin et al., 2010). In mammalian touch domes, the basal surface of the Merkel cell is innervated by slow-adapting, type 1 low threshold mechanosensory afferents (SA-LTMR) (Lumpkin et al., 2010); in addition, they make fingerlike projections between apical keratinocytes (Landmann and Halata, 1980; Figure 1A). Merkel cells make up to 3–6% of mammalian epithelia (Fradette et al., 2003), meaning there are roughly 100 Merkel cells per square millimeter of skin (Nikolaev et al., 2020). In 1875, Friedrich Merkel first characterized Merkel cells in a series of drawings that were part of his hypothesis of *zelligen enden als eigentliche tastnerven* (“cellular ends as the actual tactile nerves”), which contrasted the prevailing understanding of *freien enden dagegenals temperaturnerven* (“free ends of temperature nerves”) (Merkel, 1875). As they are now identified with a diversity of functions, there may be no single Merkel cell function (Xiao et al., 2014).

Merkel cells have been characterized in several vertebrates including birds (Watanabe et al., 1985; Toyoshima, 1993), fish and rays (Tachibana et al., 1984; Whitear, 1989), amphibians (Bani, 1982), and reptiles (Whitear, 1989). Merkel cells also make up part of highly specialized tactile organs such as Eimer’s organ of the star-nosed mole (Marasco and Catania, 2007), the push rod of the monotremes (Proske et al., 1998), the integumentary sense organ of the crocodile (Schneider et al., 2016), and the barbel of fish (Tachibana et al., 1984). In mammals, Merkel cells are found in fingers and are associated with hairs where they sense deflection (Smith, 1970; Winkelmann and Breathnach, 1973). A prime example of Merkel cells functioning as deflector sensors are in whiskers, stiff functional hairs for which deflection is important for tactile sensation in several mammals. Whiskers are an important resource for studying Merkel cells because of the ease with which experimenters may control sensory stimulation. For example, in a study where the Merkel cells of mouse hair follicles were made to express a *Clostridia* neurotoxin, the mice retained their differentiation of tactile stimuli, but reduced variation in whisker deflection was observed (Lemercier and Krieger, 2022). In humans, Merkel cells are present in both the hairy and glabrous skin; for example, they are associated with the hair of the scalp (Moll, 1994; Narisawa et al., 1994) and are important for the development of fingerprints (Polakovicova et al., 2023). In a fascinating report, Jarocka et al. (2021) showed that spatial selectivity for receptive touch fields corresponds to the dimension of fingerprint ridges which is ∼400 μm, indicating that Merkel cells detect mechanical events at individual ridges.

Merkel cells are a salient case against the “mainly neuron” paradigm of sensation. Indeed, Merkel cells themselves express the mechanosensitive channel Piezo2, and are necessary and sufficient to evoke the firing of the associated/neighboring nerve endings/low-threshold mechanoreceptors (LTMRs) (Ikeda et al., 2014; Ranade et al., 2014; Woo et al., 2014; Figure 1A). Ranade et al. (2014) characterized the behavior of Piezo2 null mice, demonstrating through the mice’s reduced responses to cotton swabbing, that their ability to respond to high forces is impaired (Maksimovic et al., 2014). Moreover, Ikeda et al. (2014) demonstrated that Piezo2 channels were the primary site of tactile transduction in rat whisker follicles, inducing Ca^2+^ potentials that drove the firing of the sensory afferent, and that they were necessary for behavioral responses to touch. Woo et al. (2014) further showed that in Merkel cell-specific Piezo2 knockout mice, the slowly adapting fibers, which are mediated by the Merkel cell-neurite complex, exhibit reduced static firing rates. In addition, these mice display decreased behavioral responses to gentle touch (Woo et al., 2014). Thus, a general model for the sensory response at touch domes has emerged in which Piezo2, as well as mechanically gated channels that are yet to be characterized, depolarize the Merkel cell membrane, induce the activation of voltage-gated Ca^2+^ channels, and cause the Ca^2+^-dependent release of neurotransmitters. Although many details remain ambiguous, several prospective aspects of this model have been identified. For example, among 362 elevated Merkel cell transcripts, L-type and P/Q-type channels appear mainly responsible for Ca^2+^ entry (Haeberle et al., 2004). However, Merkel cell Ca^2+^ transients may also be induced by Ca^2+^-induced Ca^2+^ release, given that caffeine enhances sensitivity to touch (Senok and Baumann, 1997). Remarkably, microtubules may also have an important role in potentiating the Piezo2-induced current among afferents (Chang and Gu, 2020b). The identification of the exact mechanism of Ca^2+^ entry is important for clarifying the function of Merkel cells in touch and for identifying novel targets for the treatment Merkel cell-related neuropathies; thus, more efforts in this direction may be made in the future.

Investigators have tried to unravel the identity and downstream machinery of the chemical transmitters present between Merkel cells and their neurites (Figure 1A). For example, Merkel cells of the ventral rat torso are immunoreactive to serotonin (English et al., 1992), and those of hairy skin of mice express the transcript for a vesicular glutamate transporter (Haeberle et al., 2004). In general, glutamate is the predominant somatosensory transmitter for pain and temperature (Basbaum et al., 2009). Indeed, a role for Merkel cell-derived glutamate has been identified in co-cultures of Merkel cells and trigeminal neurons (Higashikawa et al., 2019). In Higashikawa et al. (2019), mechanical stimulation of Merkel cells from hamster mucosa provoked Ca^2+^ transients in rat trigeminal neurons, while glutamate- and NMDA-receptor antagonists suppressed this activity. However, in two other reports, Chang et al. (2016, 2017) found that serotonin (5-HT), but not glutamate or norepinephrine, evoked robust impulses *in vitro* among bundles of mouse whisker-pad afferents. Further experiments revealed that the 5-HT3 receptor was important for these currents (Chang et al., 2016, 2017). Importantly, the presence of Merkel cell-derived serotonin was detected by amperometry, and the transcript for Merkel cell tryptophan hydroxylase, the serotonin synthetic enzyme, was detected by single cell RT-PCR (Chang et al., 2016).

In a competing report, Hoffman et al. (2018) utilized RNA sequencing, reverse genetics, and receptor blockade to identify adrenergic signaling from the Merkel cells of mice by way of SNARE-mediated vesicle release (Figures 1B, C). Surprisingly, in this study, no Merkel cell-derived serotonin was detected in whisker pads by HPLC, nor were transcripts for tryptophan hydroxylase significantly abundant in the RNA-sequencing of hairy dorsal skin (Hoffman et al., 2018). In their careful discussion, the authors pointed to amperometry’s lack of selectivity for the different biogenic amines and the broad expression of the 5-HT receptors as reasons for the conflicting data (Hoffman et al., 2018). Furthermore, these authors clarified that Merkel cells may indeed produce serotonin-derived currents in other species, or in mammalian pain and itch (Hoffman et al., 2018), and that there remains a role for serotonin in Piezo2-dependent mechanotransduction in the gastrointestinal tract (Kola et al., 2022).

In response, two further reports were produced in support of the serotonin hypothesis (Sonekatsu et al., 2019; Chang and Gu, 2020a). In the first report, currents in whisker Merkel disc afferents were not induced by norepinephrine and a β2 receptor antagonist had no effect on these currents at 1 μM, though there was an effect at the higher concentration of 50 μM that Hoffman et al. (2018) had employed. Therefore, these authors attributed part of the evidence for adrenergic transmission from Merkel cells to a non-specific suppression of excitability (Sonekatsu et al., 2019). In the second of these reports, sensory afferent currents were modulated by compounds which affect the release and reuptake of serotonin and were interpreted as a further line of evidence in favor of serotonergic transmission (Chang and Gu, 2020a). It seems possible that the tryptophan hydroxylase is present in the mouse whisker pad, as identified by Chang and Gu (2020a), but not in the hairy dorsal skin, as found by Hoffman et al. (2018). However, the negative finding by HPLC regarding the presence of serotonin in whisker pads, as well as the conflicting results of several experiments regarding the proposed role for norepinephrine, will need further experimentation to reconcile.

Despite progress in the last decade, significant characterization of the more intricate molecular mechanisms which connect Piezo2 activation to downstream tactile signaling and the release of neuromodulators from Merkel cells remains to be elucidated. Furthermore, whether Merkel cells exert forces upon their associated cell types (neuron, keratinocyte) via contact sites (such as tethers) remains unknown. In addition, if the Merkel cell responds to mechanical forces, what is the functional significance of the sensory afferents’ mechanosensitivity? Merkel cells are responsive to magnetic fields and other non-chemical stimuli (Xiao et al., 2014), and are activated by hypo-osmolarity (Boulais et al., 2009). How do these sensory modalities interface with the sense of touch? More models for studying the biological significance of Merkel cells are needed. Recently, a zebrafish model for studying Merkel cell biology has been developed. This model bypasses the problem that Merkel cells develop *in utero* in mammals and promises to reveal important information about Merkel cells maturation during skin organogenesis and function (Brown et al., 2023). This model, as well as the advances in Merkel cells co-culture and computational approaches, may help to illuminate further roles for Merkel cells in the sense of touch.

## Keratinocytes

In human fingertips, Merkel cells are present at their highest density of roughly 100 per square millimeter, and therefore response to gentle touch and its detection of micron-level perturbations must involve other cell types (Denda and Nakanishi, 2022). Keratinocytes, which are named for their expression of the ubiquitous structural protein keratin, make up more than 90% of the cells in the epidermis and are postulated to be touch receptors (Rook et al., 2010). As mentioned, keratinocytes make apical contact with Merkel cells in touch domes, and they are in either direct contact or are close to the terminal of all afferent subtypes of the skin (Owens and Lumpkin, 2014). Similar to Merkel cells, keratinocytes are intrinsically mechanosensitive, with their membranes depolarizing through direct mechanical stimulation (Moehring et al., 2018). Activation of keratinocytes likely leads to the release of neurotransmitters because in co-cultures of keratinocytes and dorsal root ganglion (DRG) neurons, mechanical activation of keratinocytes leads to Ca^2+^ transients in the neurons (Klusch et al., 2013). A foundational report by Baumbauer et al. (2015) utilized an optogenetics approach *in vivo* to demonstrate that depolarization of mouse keratinocytes induces action potentials in in multiple afferents, some of which are tuned to thermal sensation and nociception. In the same report, mice with keratinocytes expressing the inhibitory halorhodopsin displayed a lesser response to nociceptive stimuli when the protein was active and cell function was silenced (Baumbauer et al., 2015). Therefore, the authors concluded that keratinocytes are important for the sensation of painful touch in mice (Baumbauer et al., 2015).

**FIGURE 2:**
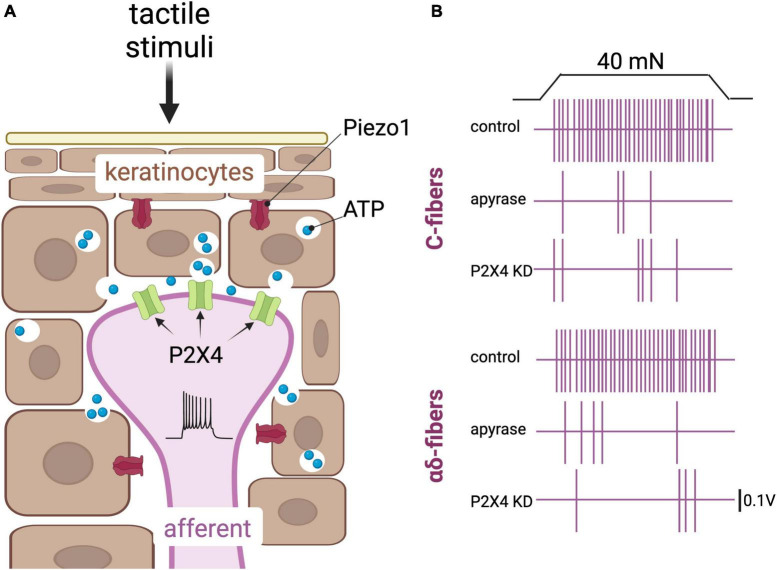
Keratinocytes are postulated touch receptors. **(A)** Depiction of keratinocytes (brown) and an afferent fiber (purple) in the epidermis. Tactile stimuli impinged on the skin activate the mechanically gated cationic channel Piezo1, leading to release of ATP from these cells and subsequent activation of the purinergic P2X4 receptors in sensory afferents (Coste et al., 2010; Baumbauer et al., 2015; Mikesell et al., 2022). **(B)** Schematic representation of key results obtained by Moehring et al. (2018) that support a role of purinergic signaling in keratinocytes to afferent communication. Top panel, a 40 nN force applied onto a mouse skin-nerve preparation induces action potentials in a C-fiber. Action potentials are depicted here as vertical purple lines. The action potentials are fewer in a skin-nerve preparation treated with the ATP hydrolyzing enzyme apyrase, and in a skin-nerve preparation from a mouse in which the P2X4 receptor was knocked down in sensory neurons using the cre/lox system and the sensory neuron-Advillin promoter. Bottom panel, same as the top panel but with an αδ fiber [adapted from Moehring et al. (2018)].

Following this work, Moehring et al. (2018) demonstrated that pain responses in mice were also dependent upon keratinocyte ATP signaling as well as upon the associated sensory-neuron receptor for ATP: P2X purinoceptor 4 (P2X4) (Figure 2). Furthermore, this group demonstrated that optogenetic inhibition of keratinocytes, and blockage of neural P2X4, altered the behavioral responses of mice when exposed to hot and cold stimuli (Sadler et al., 2020). Piezo1 was identified by these authors as the primary mechanotransducer of keratinocytes, and its deletion revealed a decrease in the mechanical sensitivity of mice, measured as paw attendant behavior (Mikesell et al., 2022). This latter report confirmed speculation from a decade earlier, when expression profiles revealed that Piezo1 was highly expressed in mouse epidermal tissue (Coste et al., 2010).

The hypothesis that keratinocytes contribute to the perception of painful touch was also found consistent with expression of voltage-sensitive channels Na(v)1.1, Na(v)1.6, and Na(v)1.8 in keratinocytes of the rat epidermis, and with detection of Na(v)1.5, Na(v)1.6, and Na(v)1.7 in human biopsies of epidermis (Zhao et al., 2008). Interestingly, biopsies from subjects with complex regional pain syndrome type 1 and post-herpetic neuralgia revealed increased markers of these proteins relative to controls (Zhao et al., 2008). Pang et al. (2015), utilizing expression of the capsaicin receptor TRPV1 under the control of the keratin 5 promoter, showed that activation of keratinocytes induced neuronal activation marker c-fos in mice and evoked paw licking and other avoidance behaviors. This result amplifies the importance of keratinocytes relevant to the sensation of painful touch in mammals (Pang et al., 2015).

In a progressive report which preceded these data, Chateau and Misery suggested that keratinocyte to neuron connections ought to be considered “synapses” proper (Chateau and Misery, 2004). For example, double immunolabeling revealed overlapping areas between keratinocyte membranes and their associated neuron and several canonical synaptic features such as accumulation of opaque material facing the post-synaptic membrane (Chateau and Misery, 2004). Along these lines, application of the gap junction blocker octanol was found to stop the propagation of intracellular Ca^2+^ ions among differentiated keratinocytes, and touch stimulation was blocked by ATP hydrolysis (Tsutsumi et al., 2009). More recently, keratinocyte-neuronal contacts in human biopsies were found to contain narrow clefts, to express synaptophysin and synaptotagmin 1, and to contain a SNARE-mediated vesicle system, all of which are molecular hallmarks of synapses (Talagas et al., 2020).

Intriguingly, the large glycoprotein laminin-332 is expressed by keratinocytes and was found important for the suppression of rapid-adapting currents in mouse DRG (Chiang et al., 2011). The authors attributed this phenomenon to the blocking of the formation of an unidentified 100 nm protein tether to sensory afferents (Chiang et al., 2011). To the best of our knowledge, the identity of this tether and its associated proteins remains unknown; however, the phenomenon of an accessory cell tethered to its associated neurite is thought to be generally relevant to touch (Chuang and Chen, 2022), and others suggest that tethers are part of a tuning element for proteins like those of the Piezo family (Richardson et al., 2022). This tethering concept will be further examined in the next section in the context of the lamellar touch corpuscles of mammals and birds.

Though keratinocytes are the primary epidermal cells in mammals, progress regarding the molecular contribution of these cells to touch has occurred mostly within the last 15 years. Further elucidation of the molecular contacts between keratinocytes and neurons may allow for the discovery of compounds which can treat pain and which are free of nervous system-mediated side effects (Owens and Lumpkin, 2014). Recent work on keratinocytes has also provoked the suggestion that biologists should move toward a “whole epidermis” view of touch, recognizing less segregation between the integumentary system and the nervous and immune systems in their respective contributions to touch and pain (Talagas, 2023). As with Merkel cells, keratinocytes are responsive to several other touch-related stimuli, including changes to atmospheric pressure, ultrasound, and magnetic fields, implying these cells may be considered diverse “information processing centers” (Denda and Nakanishi, 2022). How these other stimuli interface with the sense of touch and pain mediated by keratinocytes remains unexplored.

## The lamellar cells of the Meissner and Grandry corpuscles

There is roughly one Meissner corpuscle located within every two to four dermal papillae of mammalian glabrous skin (Piccinin et al., 2022). The lamellar cells of the Meissner tactile corpuscle are elongated Schwann-like cells with peripherally displaced nuclei (Piccinin et al., 2022). Lamellae were detailed in the first known drawings of the touch corpuscle by PhD student George Meissner and his advisor Rudolf Wagner in the mid-19th century (Wagner and Meissner, 1852). Surrounded by a CD-34 phosphoglycoprotein positive capsule, lamellar cells are perpendicular to the skin and wrap as a “coin stack” around afferents (Cobo et al., 2021; Piccinin et al., 2022). In monkeys, Meissner corpuscles may be innervated by more than one neurite (Pare et al., 2001), while in mice multiple afferents may differ in their mechanosensitive functionalities (Neubarth et al., 2020). Meissner corpuscles are responsible for the sensations of light touch, relatively low (10–50 Hz) vibrational frequencies, and slow indentation speeds up to 100 μm/ms (Simonetti et al., 1998; Piccinin et al., 2022).

**FIGURE 3:**
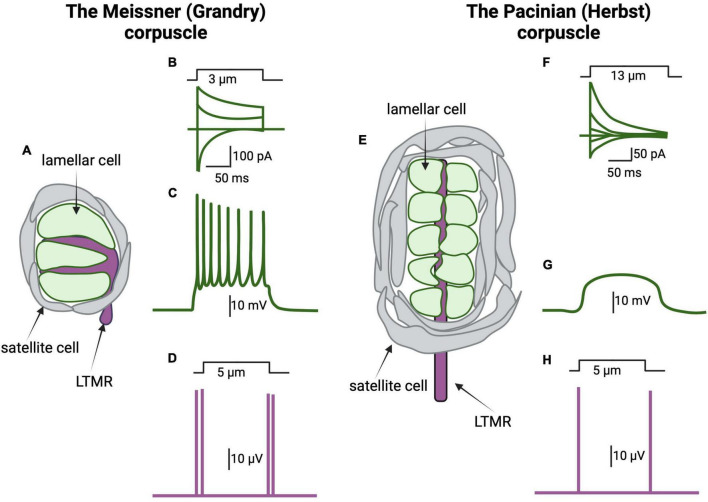
The lamellar cells of the Meissner and Pacinian corpuscles are mechanosensitive. **(A)** Schematic representation of a Grandry corpuscle found in a duck’s bill. The Grandry corpuscle in birds corresponds to the mammalian Meissner corpuscle. These corpuscles are 20–40 mm in diameter and 50–150 mm in length. The sensory afferent (purple) is surrounded by lamellar cells of Schwann cell origin (green) stacked like coins along the length of the corpuscle. A layer of satellite cells encapsulates both the lamellar cells and the afferent. **(B)** Mechanical stimulation, by application of a 3 μm indentation to the corpuscle, induces the activation of mechanically gated currents at different voltages in the lamellar cells. **(C)** The lamellar cells of the Grandry corpuscle express voltage gated Ca^2+^ and K^+^ channels and thus are capable of generating action potentials following stimulation with current injection or mechanical forces. **(D)** Schematic representation of afferent response to mechanical stimulation of the Grandry corpuscle. The afferent responds to application and removal of the mechanical stimulus by action potential firing (purple lines). The response is rapidly adapting; thus, no action potential is seen during the static portion of the indentation. **(E)** Schematic representation of the Herbst corpuscle in ducks. Herbst corpuscles are also found in the duck’s bill alongside the Grandy corpuscles. Herbst corpuscles in ducks are similar in size to Grandry corpuscles. However, the corresponding mammalian touch receptor, the Pacinian corpuscle, can reach 1 mm in length. **(F)** Mechanical stimulation of the Herbst corpuscle induces the activation of mechanically gated currents at different voltages in the lamellar cells. Note that the kinetics of the Herbst’s mechanically gated currents are different than those of the mechanically gated currents recorded from the Grandry corpuscles, suggesting differences in the underlying mechanosensitive channels. **(G)** The lamellar cells of the Herbst corpuscles do not express voltage-gated ion channels; thus, these cells do not fire action potentials when stimulated. **(H)** Herbst corpuscles are also rapidly adapting, so their afferents respond to mechanical stimulation by firing action potentials during application and removal of the mechanical stimulation [adapted from Nikolaev et al. (2020), Ziolkowski et al. (2022)].

In recent years, functional studies of Meissner corpuscles in birds like the duck have been published. The duck, a tactile forager, relies on the sense of touch when locating and filtering food, particularly when submerged in water or mud (Matos-Cruz et al., 2017). In birds, the Grandry corpuscle of the beak is functionally and structurally similar to Meissner’s corpuscle, and recent publications may thus use “Meissner” in place of “Grandry” (Gottschaldt, 1974; Nikolaev et al., 2020). Fittingly, the Grandry corpuscle was first described by Grandry (1869), Theodor Schwann’s pupil, more than a decade after the report of Meissner and Wagner. These avian models provide two main advantages: first, their beaks are large and filled with Meissner corpuscles (65/mm^2^), and second, the avian somatosensory system is largely complete before hatching, allowing for experiments to be performed in experimentally more accessible embryos instead of full-grown birds (Berkhoudt, 1979; Ziolkowski et al., 2022). The avian corpuscular structure is also surrounded by Schwann-derived satellite cells (Ide and Munger, 1978; Figure 3A), though no role in the sense of touch has yet been established for these cells.

In a landmark study, Nikolaev et al. (2020) demonstrated that mechanical stimulation triggers R-type voltage-gated Ca^2+^ channels-dependent action potentials in the lamellar cells of the Meissner (Grandry) corpuscle, the first evidence for the mechanically induced excitability of these accessory cells and the first demonstration of R-type channel-dependent firing in a non-neuronal cell type. In that study, the current produced by mechanical stimulation of the Meissner corpuscles, which presumably depolarizes the cell and leads to activation of the Ca^2+^ channels, displayed fast activation kinetics that are similar to Piezo2’s currents (Figures 3A–C). However, it remains unknown whether the observed current is mediated by Piezo2 or by other proteins (Nikolaev et al., 2020). Transcriptome analysis by the authors identified several putative mechanoreceptors including Piezo1 and Piezo2, transmembrane channel-like protein 2 (TMC2), and transmembrane protein 63 (Tmem63) (Nikolaev et al., 2020). The authors also reported the presence of dense core vesicles in Meissner lamellae, described previously by Watanabe et al. (1985), and suggested that these might be involved in the release of neuropeptides or other neuromodulators. Similarly, 10 nm diameter intramembranous particles of unknown function have been described in the Meissner corpuscle of mice by freeze-fracture. These particles are at a density of roughly 3,000 per square micron of lamellar cell plasma membrane (Ide et al., 1985). Taken together, these studies support the idea that the lamellar cells of the Meissner (Grandry) corpuscle are mechanosensitive and might be more directly responsible for mediating touch sensation than previously suspected, perhaps via activation of the nerve fibers by neuromodulators.

More recently, Nikolaev et al. (2023) established that the lamellar cells of the Meissner corpuscle are indeed touch sensors. Utilizing scanning electron microscopy and electron tomography, these authors modeled a three-dimensional architecture of duck bill corpuscles, revealing the dense core vesicles as well as tether-like connections between lamellar cells and afferent membranes (Nikolaev et al., 2023). In this report, electrophysiological recordings revealed that Ca^2+^ influx among lamellar cells preceded action potentials of the associated afferent (Figure 3C), and that these phenomena are disjoint and therefore prohibitive of direct electrical coupling (Nikolaev et al., 2023). The authors hypothesized a role for chemical transmission by exocytosis, from lamellae to afferent, in part because removal of extracellular calcium suppressed mechanically induced action potentials in the afferents. Finally, a “bi-cellular” mechanism for touch detection in the Meissner corpuscle was proposed. In this mechanism, afferent Piezo2 directly mediates initial responses while lamellar cells contribute through an unknown secondary and complementary mechanism that may involve chemical signaling or physical contact (Nikolaev et al., 2023). This type of model may allow for a versatile range of touch perception that is crucial to the complex foraging behaviors of ducks, and to the high capacity for object manipulation of humans and of other mammals (Nikolaev et al., 2023).

Over a decade ago, a further role for tether proteins in mechanotransduction was proposed (Hu et al., 2010), and this proposal has proven fruitful regarding the mechanism of touch in the Meissner corpuscles. Usherin type 2A (USH2A) is a transmembrane protein proposed to form tethers at hair-cell stereocilia (Adato et al., 2005). Mutations in the USH2A gene are a frequent cause of Usher’s syndrome, a disease which affects hearing and vision in humans (Eudy et al., 1998). Schwaller et al. (2021) combined human genetic resources and murine biochemistry to elucidate the role of USH2A in mechanotransduction. The authors reported that patients with loss of function mutations in USH2A displayed reduced perception of 10 and 125 Hz vibrations (Schwaller et al., 2021). Furthermore, in a vibration learning task, USH2A null mice had decreased performance when exposed to 5 and 25 Hz vibrations. Strikingly, the expression of USH2A was localized to Meissner lamellae. These authors clarified that their exciting result does not prove there is an intercell tether complex between the lamellae and the neurite, and that details of the exact site of touch reception remains mysterious (Schwaller et al., 2021). Intriguingly, Handler et al. (2023), using high-resolution enhanced Focused Ion Beam Scanning Electron Microscopy (FIB-SEM), recently reported an extensive network of interdigitations between neuronal terminal protrusions/spines and lamellar cells’ caveolae-like invaginations in the Meissner and Pacinian corpuscles as well as in the hair follicle. Adherens junctions and Piezo2 channels are localized at these interdigitations (Handler et al., 2023). It is thus tempting to speculate that molecular tethers linking nerve terminals and accessory cells might be present at these locations.

The laboratory of José Vega has also added important insights into the structure and function of the Meissner’s lamellae. For example, they showed that lamellar cells express the brain-derived tropomyosin receptor kinase b (TrkB) and the acid-sensing ion channel ASIC2, a member of the DEG/ENaC family of channels implicated in touch sensation in worms, flies, and mice (Huang and Chalfie, 1994; Price et al., 2000; Calavia et al., 2010a; Zhong et al., 2010; Cabo et al., 2015). This group also reported the presence of the transient receptor potential channel TRPV4 in the lamellar cells, a channel involved in hyperalgesia (Alonso-Gonzalez et al., 2017). In their study in Meissner corpuscles, Piezo2 was reported in the axon but not in the lamellar cells (García-Mesa et al., 2017). Garcia-Piqueras et al. (2019) have also characterized proteoglycans, but not chondroitin sulfates, in the basement membrane of lamellar cells of Meissner’s corpuscle. The functional significance of this remains to be known, but heparin sulfate markers colocalized with type IV collagen and intercellular collagen may be important for mechanotransduction in this structure (Garcia-Piqueras et al., 2020; Piccinin et al., 2022). It is currently not known to what extent lamellar cells of the Meissner corpuscles secrete and maintain the intercellular milieu or its functional significance in touch.

## Lamellar cells of the Pacinian and Herbst corpuscles

The Pacinian corpuscles were described several times in the 18th century but their name comes from the 1835 communications of medical student Filippo Pacini to the medical society of Florence (Bentivoglio and Pacini, 1995). Pacinian corpuscles and their lamellae are described in the deep dermis of several mammals; however, in mice they are found mostly in the periosteum of some bones (Handler and Ginty, 2021). They have been characterized in the glabrous skin of several mammals (Luo et al., 2009), in the epidermis of amphibians (von During and Seiler, 1974), and in reptiles (Leitch and Catania, 2012). The presence of Pacinian corpuscles in the foot is presumably related to the surprising phenomenon of seismic communication, by which elephants can sense vibrations at distances up to 30 km (O’Connell-Rodwell, 2007). Quindlen-Hotek et al. (2020) have assembled a helpful list of the known reports and dimensions of Pacinian corpuscles and lamellar-like touch sensors among vertebrates.

In humans and mice, Pacinian corpuscles are responsible for the sensations of high (20 Hz to 10 kHz) frequencies of vibration and of faster indentation speeds up to 400 μm/ms (Simonetti et al., 1998; Quindlen et al., 2016). They are composed of lamellar cells of Schwann cell-origin encapsulating a rapidly adapting, type 2 sensory afferent (Cauna and Mannan, 1958; Cobo et al., 2021; Figure 3D). In addition, a capsule of connective tissue forms the outer most layer that separates the corpuscle from the surrounding tissue. Early studies done in cats showed that manual removal of the capsule and lamellae prolongs the generator potential, suggesting an important function of the lamellae in touch transduction (Loewenstein and Mendelson, 1965).

Just as the Grandry corpuscle of birds is analogous to the mammalian Meissner, the Herbst corpuscle of birds is functionally and structurally like the Pacinian (Gottschaldt, 1974). The Herbst corpuscle was named for its discoverer, the German physiologist Curt Alfred Herbst, and is found in the bill skin of tactile foragers and of remote-sensing foragers like the kiwi (Martin, 2017). In ducks and geese, there are an exceptionally large number of these sensors, with up to 140 Herbst corpuscles per square millimeter (Gottschaldt and Lausmann, 1974; Berkhoudt, 1979; Watanabe et al., 1985). For perspective, there are roughly 300 Pacinian corpuscles in the human hand (Stark et al., 1998). The Herbst corpuscle is present near the Grandy corpuscles and in similar numbers, though the former is nearly twice the diameter (Avilova et al., 2018). Herbst corpuscles are also found in the footpads of some birds such as the parrot (Lennerstedt, 1975), where they are presumably relevant to the careful branch movements and manipulation of food and objects in this species (Demery et al., 2011).

In the same report mentioned above, Nikolaev et al. (2020) demonstrated that the outer core of Pacinian (Herbst) corpuscles is mechanosensitive. However, contrary to the Meissner corpuscle, the lamellae of Pacinian (Herbst) corpuscles are not capable of generating action potentials (Nikolaev et al., 2020; Figures 3E–H). In this study, Pacinian and Meissner activation kinetics were found to be different from each other, as the decay kinetics of the Pacinian were slow compared to Piezo2 currents. This suggests that either other channels mediate the mechanosensory currents in the lamellae of the Pacinian, or that Piezo2 currents are modified in these cells by accessory proteins or cellular signaling (Nikolaev et al., 2020).

**FIGURE 4:**
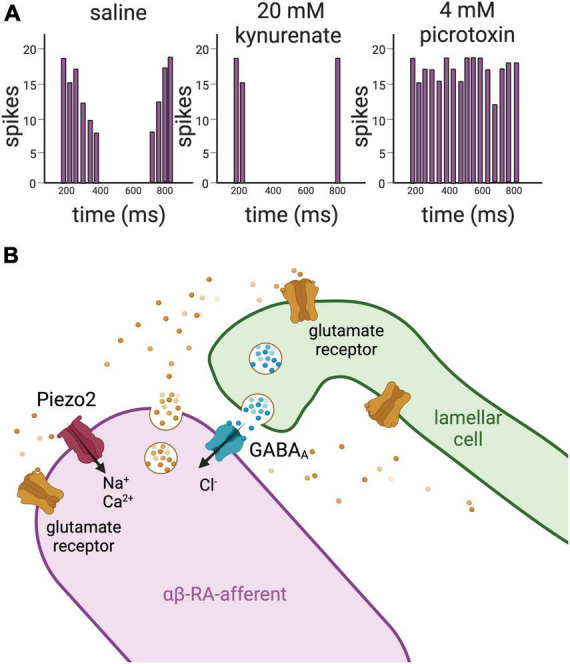
Glutamate and GABA mediate the crosstalk between the lamellar cells and the afferent in Pacinian corpuscles. **(A)** Left panel, schematic representation of action potentials’ firing recorded from a LTMR of a Pacinian corpuscle isolated from a cat upon application of mechanical stimulation. The afferent fires action potentials upon application and removal of the mechanical stimulation, but not during the static portion of the indentation. Middle panel, upon application of the glutamate receptor antagonist kynurenate, there is a significant loss of activity in the afferent. Left panel, on the contrary, addition of the GABA_A_ receptor antagonist picrotoxin induces increase of firing of action potentials in the afferent. Together these data support that glutamate and GABA act on the LTMRs of Pacinian corpuscles to regulate their excitability. **(B)** Drawing representing glutamate and GABA signaling between the lamellar cells and the afferent in Pacinian corpuscles based on results obtained in the cat (Pawson et al., 2000, 2007, 2009). RT-PCR demonstrates the presence of the vesicle-associated protein VAMP2 in the lamellar cells, suggesting that the lamellae of the Pacinian corpuscles are capable of establishing a synapse-like structure with the afferent. This idea is supported by the detection of clear core vesicles within the lamellae. Clear core vesicles are also detected in the afferent. In addition, antibody staining detects glutamate receptors and GABA_A_ receptors in the innermost core of the Pacinian corpuscle, where the afferent makes contact with the lamellar cells. Piezo2 channels have been recently localized to the αβ-RA-afferents of the Pacinian corpuscles of the mouse (Handler et al., 2023).

Pawson et al. (2000) have also made exciting progress on the role of Pacinian lamellae in mechanotransduction. Extensions of the Pacinian neurite, called filopodia, were observed to contain a high density of actin, reminiscent of stereocilia in hair cells and perhaps similar in their mechanoreceptive quality (Pawson et al., 2000). In a landmark study, these authors reported immunoreactivity for GABA receptors in the Pacinian afferent, gene expression of synaptobrevin in lamellae, and the appearance and disappearance, respectively, of action potentials in the Pacinian neurite in the presence of GABA and of GABA receptor antagonists gabazine or picrotoxin. These data, together with the ablation of currents by the glutamate blocker kynurenate, were interpreted as a “mechanochemical, rather than purely mechanical” response of rapid adaptation during the static portion of sustained pressure (Pawson et al., 2007, 2009; Figure 4).

Regarding protein expression that is relevant to mechanosensation, the laboratory of José Vega detected the DEG/ENaC acid-sensing channel ASCI2 in some but not all of the Pacinian inner core lamellae of the mouse (Montano et al., 2009; Calavia et al., 2010b). In addition, they showed that β-ENaC and γ-ENaC, other members of the DEG/ENaC channel family, can be detected in the inner core lamellae of neurotrophin 4 deficient mice (Montano et al., 2009). While the importance of this finding remains to be clarified, expression of these channels appears to be a common feature of accessory cells, such as the Schwann-related cells of rat Ruffini endings (Hitomi et al., 2009), and the satellite cells of the rat DRG (Kawamata et al., 2006). Some contrasting results concern the expression of voltage-gated ion channels by the Pacinian lamellae. While Pawson and Bolanowski (2002), using immunocytochemistry, reported the expression of type I and II voltage-gated sodium channels in the lamellae, Nikolaev et al. (2020) did not find that these cells were excitable, at least in the duck bill and under their experimental conditions (Nikolaev et al., 2020). Thus, it remains to be determined whether voltage-gated Na^+^ channels have a function in the lamellar cells of the Pacinian corpuscle.

The question of mechanotransduction at the Pacinian corpuscle has long been thought to be amenable to modeling. For example, early models indicated that fluid-lamellar interaction may act as a band-pass filter of vibrations sensed by the neurite (Loewenstein and Skalak, 1966). Quindlen et al. (2016) developed the first multistage model for the Pacinian corpuscle. In their model, the speed of adaptation, depth, and shape of lamellar cells contributed greatly to touch sensitivity; however, the authors acknowledged that neurotransmitter signaling and a greater diversity of ion channels should be included in future work. In a second study, the computational approach of these authors was applied to the hypothesis that broad generalities exist among the Pacinian corpuscles of different vertebrates, given the observed diversity of lamellar size and layering geometry. The authors concluded that despite the large variety of sizes of the Pacinian corpuscles, nearly all of the 19 species studied showed very similar sensitivity ranges, with the only exceptions being humans and geese who are tuned to 130–170 vs. 40–50 Hz frequencies (Quindlen-Hotek et al., 2020).

These reports on the accessory cells of the lamellar (Meissner and Pacinian) touch corpuscles have provoked several questions and directions. For example, lamellar cells of the Pacinian (Herbst) corpuscle lack voltage-gated currents and are far from the neuron (Nikolaev et al., 2020). Thus, the functional significance of the mechanosensitive currents in these corpuscles remains unclear (Nikolaev et al., 2020). Furthermore, it is unknown whether synapse-like structures are in the nerve terminal or the lamellae, or both (Pawson et al., 2009). Finally, the phenomenon of rapid adaptation, though it seems at least partly mediated by GABA, is still not fully understood (Pawson et al., 2009).

## The glia of *C. elegans* touch receptors

*C. elegans* has a simple nervous system composed of 302 neurons and 56 glia, for which interactions are thought to represent the ancestral and fundamental roles for glia in neural tissue (Singhvi and Shaham, 2019). Of the 302 neurons, 30 are putative mechanoreceptors in hermaphrodites and males. In addition, there are another 52 male-specific neurons that are postulated to be mechanosensory; most of these neurons have a role in mating behavior (Goodman, 2006). Twenty-four of the nematode hermaphrodite mechanosensory neurons are associated with sheath and socket glia. These are glial cells that extend cellular processes along the sensory neurons’ dendrites and ensheath the most distal part where the dendrites form primary sensory cilia (Bae and Barr, 2008). Our lab has been exploiting *C. elegans*’ genetic amenability to advance our understanding of the role of glia associated with mechanosensory neurons in the process of touch sensation.

We reported that glia associated with OLQ (4 neurons) and IL1 (6 neurons) nose/head touch sensory neurons express the DEG/ENaC channels *delm-1* and *delm-2*, and that touch and foraging behavior are disrupted in null mutants of these proteins (Han et al., 2013). Rescue of the *delm-1* null phenotype by expression of the inward rectifier potassium channel *irk-2* in glia or a cationic channel in OLQ touch neurons suggest that basal neuronal excitability is set by the glial *delm* channels (Han et al., 2013). However, the *delm* knockout mechanosensory phenotypes are not due to changes in neuronal structure or development because rescue can be also achieved in adults by expression of the temperature-sensitive mosquito TRPA1 channel and by performing experiments at a temperature that activates TRPA1 (Wang and Bianchi, 2020). In addition to the *delm* channels, the Na^+^/K^+^ pump genes *eat-6* and *catp-1* are also needed in OLQ and IL1 glia for nose touch responses (Johnson et al., 2020). Since DEG/ENaC channels and the Na^+^/K^+^ pump are involved in controlling the homeostasis of extracellular Na^+^ and K^+^ across species, these data suggest that one of the functions of glia associated with mechanosensory neurons might be regulating the ionic composition in the shared microenvironment between neurons and glia (Johnson et al., 2020). More specifically, a role for extracellular K^+^ regulation was hypothesized because neuronal excitability is tightly dependent on extracellular K^+^. Since *delm-1* is open at baseline, it is plausible that this protein favors K^+^ excretion, thereby establishing a relatively high level of neuronal excitability significant to the touch responses. If true, this process could be broadly relevant to other neural pathways of *C. elegans* and other organisms.

**FIGURE 5:**
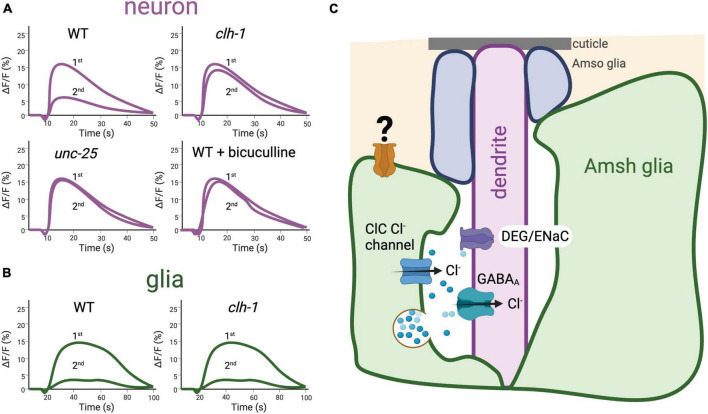
The ClC Cl^–^ channel *clh-1* is needed in the Amphid Sheath (Amsh) glia of *C. elegans* for response to nose touch by mediating GABA signaling. **(A)** Schematic representation of nose touch response in *C. elegans* ASH neurons. Using the Ca^2+^ sensor GCaMP-6s, in Fernandez-Abascal et al. (2022) we showed that nose touch stimulation induces Ca^2+^ transients (shown as change in GCaMP6s fluorescence over the baseline, ΔF/F,%) in the ASH nose touch sensory neuron mediated by the activation of DEG/ENaC channel *deg-1*. Stimulation with a second touch induces reduced Ca^2+^ transients, indicating that adaptation to touch occurs in this nematode receptor. Adaptation is prevented by knockout of the glial channel *clh-1* or of *unc-25*, the GABA synthetic enzyme glutamate decarboxylase, as well as treatment with the GABA_A_ receptor antagonist bicuculline. These data, together with others presented in Fernandez-Abascal et al. (2022) support the idea that *clh-1* is needed for glia-to-neuron GABA signaling that mediates neuronal adaptation. ASH neurons’ adaptation is in turn needed for behavioral response to nose touch. **(B)** Schematic representation of nose touch response in *C. elegans* Amsh glia. Nose touch induces Ca^2+^ transients also in the Amsh glia, indicating that these cells are mechanosensitive. The Ca^2+^ transients elicited by nose touch in Amsh glia are not dependent on *clh-1*. **(C)** Depiction of glia-to-neuron cross talk in the nose touch receptors of *C. elegans*. Longitudinal section of the Amphid sensory organ of *C. elegans* showing the Amsh glia (green) wrapping around the terminal dendrites of 12 pairs of amphid sensory neurons; here only the ASH touch neuron’s dendrite is shown for simplicity. In addition, glial Amphid Socket cells (Amso, in blue) wrap around the most distal part of the sensory dendrites. In Amsh glia, *clh-1* is needed for glia-to-neuron GABA signaling by providing the Cl^–^ ions that permeate through the neuronal GABA_A_ receptor. GABA is postulated to be released via vesicle fusion, though other modes of GABA release may exist in Amsh glia, including bestrophin channels (Wang et al., 2022). A yet to be identified mechanosensitive channel (depicted here in orange) is expressed in Amsh glial cells and is likely responsible for these cells’ mechanosensitivity [adapted from Fernandez-Abascal et al. (2022)].

We recently provided further evidence that regulation of touch neurons’ output is controlled by extracellular ions. In Fernandez-Abascal et al. (2022), we showed that *clh-1*, an inward rectifier Cl^–^ channel expressed in the Amphid Sheath (Amsh) glia, facilitates touch response in *C. elegans* via mediation of Cl^–^ efflux and, consequently, GABA receptor-dependent alteration of neuronal levels of Ca^2+^ and cyclic-AMP (cAMP) (Grant et al., 2015; Figure 5A). Rescue of the *clh-1* null touch-insensitive phenotype by the rat homolog ClC-2, a channel also expressed in glia, underscores the conservation of function for these proteins across species (Blanz et al., 2007; Depienne et al., 2013; Fernandez-Abascal et al., 2022). The broad relevance of these data is supported by the fact that mechanical hyperalgesia, in which exaggerated pain is caused by stimuli that normally elicit low pain, and allodynia, in which pain is caused by innocuous stimuli, are linked to hyperexcitability through increased cAMP/PKA signaling (Lolignier et al., 2015). Furthermore, GABA and cAMP signaling pathways have been reported in mammalian receptors for both touch and pain (Pawson et al., 2009; Zhu et al., 2014). Interestingly, we found that different sensory functions in *C. elegans* require specific regulators of ion and solute homeostasis, and that glial ablation exerted global effects beyond the loss of individual regulators (Wang et al., 2022). For example, mutants with defects in their sensation of tastants and odorants were normal in their sensation of touch, and *clh-1* touch insensitive mutants were normal in their sensation of odorants and their tolerance for high osmolarity (Fernandez-Abascal and Bianchi, 2022; Fernandez-Abascal et al., 2022). These data imply that *clh-1* and its downstream effectors are needed specifically for their role in touch (Fernandez-Abascal and Bianchi, 2022). Taken together these findings support the idea that the glia of touch receptors in *C. elegans* regulate the ionic composition of the microenvironment between glia and sensory endings and release neuromodulators such as GABA that are needed for touch responses. These mechanisms appear conserved across species. Indeed, touch receptor excitability in the Pacinian corpuscle was found to scale with ion concentration in the experimental medium (Ilyinsky et al., 1976), and regulation of extracellular K^+^ by support cells is found in Johnston’s organ and other touch receptors in *Drosophila* (see below). In addition, GABA has been reported to regulate the afferent in Pacinian corpuscles, as described above (Pawson et al., 2009). Finally, we reported that glia of *C. elegans* touch receptors are mechanosensitive, like the lamellar cells of the Meissner and Pacinian corpuscles, though this intrinsic mechanosensitivity is independent from *clh-1* and is currently of unknown function (Figures 5B, C; Fernandez-Abascal et al., 2022).

## The accessory cells of touch receptors in *Drosophila*

**FIGURE 6:**
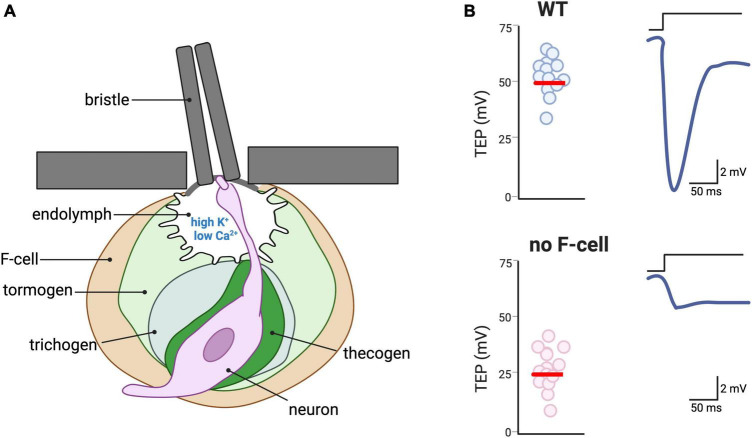
Accessory cells of touch receptors in *Drosophila*. **(A)** Depiction of a bristle, the major type of touch receptor in the adult of *Drosophila*. The hair shaft of the bristle is connected to the dendrite of a sensory neuron via physical contact, thus transducing the hair deflection into activation of mechanosensitive channels in the neuron. The dendrite of the neuron bathes in an endolymph characterized by a high concentration of K^+^ and a low concentration of Ca^2+^ (Grunert and Gnatzy, 1987; Eberl, 1999; Roy et al., 2013). The sensory neuron is surrounded by four accessory cells: the thecogen, the trichogen, the tomogen, and the F-cell, which is of epidermal origin. **(B)** Schematic depiction of the need for F-cells for response to bristle deflection. Due to the high K^+^ concentration of the endolymph, the transepithelial potential (TEP) is normally ∼ + 50 mV (**left** panel). Upon hair deflection, the activation of the mechanosensitive channel in the neuron induces repolarization of the TEP. In *Drosophila* mutants in which the F-cells are either genetically ablated or do not develop, TEP is ∼ + 25 mV and deflection of the hair shaft causes smaller changes in the TEP [adapted from Mangione et al. (2023)].

*Drosophila* has a number of touch sensilla including the bristles, the auditory receptors for mating, the wing strain gauges to respond to flight path impediments, and the proprioceptors to coordinate movement and positioning of the legs. Among these, the bristles cover the entire body of the adult fly and are the major touch receptor. The structure of the bristles resembles that of gustatory and olfactory sense organs (Stocker, 1994). All these organs consist of one or a few sensory neurons innervating a structure formed by three specialized support cells called the thecogen, the tormogen, and the trichogen (also known as the sheath and socket cells, and shaft, respectively) (Figure 6A; Keil, 1997a). The same trio that detects sound vibrations in Johnston’s organ is composed of a ligament, scolopale, and cap (Prelic et al., 2021). The extracellular space between the sensory neurons and the support cells in these sensory organs is filled with an uncharacteristically high K^+^ and low Ca^2+^ endolymph secreted by the accessory cells, which is reminiscent in its composition of the vertebrate inner ear endolymph (Grunert and Gnatzy, 1987; Eberl, 1999; Roy et al., 2013; Figure 6A). The high concentration of K^+^ in the endolymph establishes a positive transepithelial potential (Kernan et al., 1994; Keil, 1997b; Mangione et al., 2023). Upon deflection of the hair shaft, a mechanically gated current, mainly mediated by K^+^ ions, is elicited in the sensory neuron dendrite leading to a reduction in the transepithelial potential (Kernan et al., 1994). Because of the steep K^+^ gradient, the latency of mechanotransduction is only 0.1 ms (Walker et al., 2000). Thus, this mechanism of mechanotransduction heavily relies on the function of the accessory cells that must pump K^+^ into the endolymph (Roy et al., 2013). Together with the observation of K^+^-rich fluid surrounding the hair cells of mammalian cochlea (Wangemann, 2006), work in *C. elegans* touch receptors suggests that higher than normal K^+^ concentrations in the microenvironment between sensory neurons and accessory cells may not be a feature of insects only (Han et al., 2013; Johnson et al., 2020; Wang and Bianchi, 2020). The genetic amenability of *C. elegans* and *Drosophila* combined with newly developed genetically encoded ion sensors should shed some light on the mechanism that regulates the concentration of K^+^ and other ions in these touch receptors and their significance to touch (Shen et al., 2019; Zhang et al., 2023).

In addition to secreting K^+^ into the endolymph, the supporting cells of sensory organs in insects may play a more active role in the response to sensory cues. For example, Prelic et al. (2021), using the Ca^2+^ and K^+^ indicators, showed that the tormogen cells of the antenna olfactory receptors of *Drosophila* undergo changes in intracellular Ca^2+^ and K^+^ during exposure to odorants. Interestingly, the authors found no changes in intracellular Ca^2+^ and a much smaller change in K^+^ concentration in thecogen cells, as opposed to tormogen cells, upon exposure to odorants. These results suggest that these two types of support cells might have distinct functions in these sensory organs. As confirmation that thecogen cells are needed for olfaction, Prelic et al. (2021) found that the expression of the apoptotic protein reaper in these cells led to an altered response by the sensory neurons to a battery of odorants. Remarkably, the ablation of thecogen cells was also associated with an unexpected increase in mechanosensitivity (after “empty” blows of air during olfaction experiments), suggesting a conserved role for thecogen cells across different sensory organs (Prelic et al., 2021). Interestingly, a similar parallelism between the function of accessory glia in touch and olfaction was found in *C. elegans* (Duan et al., 2020; Fernandez-Abascal et al., 2022). However, there seems to be some sensory-dependent specialization because not all the glial genes required for olfaction are needed for touch and vice versa (Wang et al., 2022).

A recent publication highlights the functional role in touch of another type of cell associated with the Drosophila bristle. Mangione et al. (2023) reported about a previously undescribed epidermal cells that they named F-Cell (Figure 6A). The F-cell differentiates to acquire a specialized morphology that allows for the ensheathing of each bristle. Interestingly, the selection of the F-cell to become the bristle ensheathing cells occurs via the epidermal growth factor receptor (EGFR) pathway, with the shaft cells releasing EGF and the F-cell expressing the EGF receptor. Importantly, using electrophysiology, the authors show that the F-cell influences the neurophysiological signature of the bristle. More specifically, genetic ablation or loss of differentiation of the F-cell leads to a strongly reduced transepithelial potential upon mechanical stimulation (Figure 6B). The authors do not further investigate the mechanism but suggest that it might be mechanical, electrochemical or both. Thus, the F-cell might mediate physical coupling between the hair deflection and the neuronal depolarization, or it might modulate the ionic composition of the microenvironment surrounding the neuron (Mangione et al., 2023). Future experiments using genetically encoded ion sensors might help address this question.

The *Drosophila* larva does not have bristles, but it senses mechanical impingement on its body via type IV mechanosensory neurons imbedded in its skin. These mechanosensory neurons seem to be aided in their function by epidermal cells. Indeed, the laboratory of Jay Parrish found that disrupting the epidermal ensheathment processes of type IV sensory dendrites, which end in the recruitment of accessory and neural junction proteins, alters the nociceptive behaviors of rolling and crawling (Jiang et al., 2019). This finding is reminiscent of the discovery that keratinocytes in mammals respond to mechanical forces and thus suggests that basic principles of interaction between nerve endings and skin cells might be conserved across species.

## Multicellular contributions to touch in plants

Among plants, there exists a diverse set of responses to touch. For example, *Dionaea muscipula*, commonly known as the Venus fly trap, closes tightly upon touch; this allows for the trapping and consuming of insects, which contributes to its ability to thrive in nitrogen-poor environments (Braam, 2005). The model plant *Arabidopsis thaliana* can sense caterpillar vibrations and discriminate these movements from vibrations caused by wind or insect sounds (Appel and Cocroft, 2014). Furthermore, an *Arabidopsis* knockout of Piezo1 was observed to have impaired root growth into hard media, implying that the function of this protein is also conserved among plants (Mousavi et al., 2021).

There is evidence that accessory cells to primary mechanotransducers exist in plants as well. For example, in *Mimosa pudica*, a creeping shrub of the pea subfamily, light touch causes the striking closure of all leaflets. *Mimosa* is therefore known by the conventional names “sensitive plant” and “touch-me-not.” The main cause of leaflet closure is changes in turgor pressure in the pulvinus, the enlarged segment at the base of the leaf stalk (Braam, 2005). Thus, the pulvinus is thought to be the main site of mechanosensation. However, Tran et al. (2021) demonstrated that leaflet closure in *Mimosa* is attenuated by the application of a known blocker of mechanically gated channels GsMTx4 in cells proximal to the pulvinus, rather than in the pulvinus itself. These data suggest that these nearby cells may be accessory cells that play an important role in mechanosensation.

Other cells with potential roles as accessory cells in mechanotransduction were identified by Visnovitz et al. (2007). By microscopy, these investigators identified previously unknown “red cells” containing high amounts of the polyphenol tannin in vacuoles, which are connected to motor regions of the *Mimosa* pulvini by plasmodesmata—threads of cytoplasm which pass through adjacent plant cells (Visnovitz et al., 2007). Electrophysiological measurements of these cells revealed them to be excitable (Visnovitz et al., 2007), and the progenitors of these red cells, the stomatal subsidiary cells, are known to mediate the opening and closing of stomatal pores and influence K^+^ and Cl^–^ flux in their surroundings (Raschke and Fellows, 1971). This mechanism is reminiscent of the regulation of ion concentrations in the touch receptors of animals, as discussed earlier. Furthermore, in *Mimosa*, application of the K^+^ ionophore valinomycin and the K^+^ blocker tetraethylammonium chloride to the pulvinus blocked leaflet closure. However, the gravitropic movement of leaflets was unaffected, and was more strongly blocked by the ablation of other ionic gradients (Roblin and Fleurat-Lessard, 1987). Also, the measure of action potentials in excitable cells of the pulvinus varies with the external concentration of Cl^–^ (Samejima and Sibaoka, 1982), and as extracellular Cl^–^ concentration increases during leaflet movement, concentrations of Cl^–^ and K^+^ appear to be exchanged between the various cell-types of the pulvinus (Hagihara and Toyota, 2020). The parenchyma of the phloem also contains excitable cells (Sibaoka, 1962), and unloading of phloem sucrose is thought to be a major factor in the observed turgor pressure changes (Fromm, 1991). To conclude, control of shared milieu appears to be important for closure of *Mimosa*, with several of these potential accessory cells (red cells, parenchymal, etc.) receiving signals, becoming excited, and contributing to idiosyncratic aspects of the shared space.

## Conclusion and future directions

Provoked by the work of disparate laboratories, some broad considerations about the accessory cells of touch receptors have emerged. First, there is the convergent idea from multiple systems that the maintenance of unique ionic concentrations may be important for the sense of touch. In particular, the concentration of K^+^ in the environment between mechanosensory neurons and accessory cells is essential for touch transduction and established by accessory cells (Raschke and Fellows, 1971; Grunert and Gnatzy, 1987; Eberl, 1999; Wang et al., 2008, 2022; Han et al., 2013; Roy et al., 2013; Johnson et al., 2020; Prelic et al., 2021; Mangione et al., 2023). The concentration of Cl^–^ ions also seems to be tightly regulated and may be important for accessory cells-to-sensory afferents’ GABA signaling (Samejima and Sibaoka, 1982; Hagihara and Toyota, 2020; Fernandez-Abascal et al., 2022; Wang et al., 2022). The development of genetically encoded ion sensors capable of reporting on extracellular ion concentrations, rather than intracellular, will be crucial for advancing our understanding of how accessory cells contribute to the ionic composition of the microenvironment surrounding nerve endings across various types of receptors and species. Second, accessory cells of touch receptors release neurotransmitters including GABA, glutamate, serotonin, and adrenaline. In the future, it will be important to determine whether different types of touch receptors release different neurotransmitters, and/or whether different neurotransmitters are released depending on the type of stimulation or condition. Furthermore, the potential role of neuropeptides, normally present in dense core vesicles described in at least some touch receptors such as the Meissner and Pacinian corpuscles, should be investigated as they may provide more long-term regulation of the excitability of the afferents (Watanabe et al., 1985; Abraham and Mathew, 2019; Nikolaev et al., 2020). Third, the physical interaction between accessory cells and nerve ending should be further explored. The network of interdigitations between the sensory neurons’ spines and the invaginations on the Schwann cells in the Meissner and Pacinian corpuscles, as well as in the hair shafts, is striking (Handler et al., 2023). This, combined with the strategic localization of the Piezo2 channels at these locations, suggests physical contact, perhaps via tethers. Such physical contact may facilitate the opening of mechanically gated channels and/or the release of chemical transmitters upon application of mechanical forces (Mangione et al., 2023; Nikolaev et al., 2023).

Finally, one of the biggest mysteries about the accessory cells of touch receptors is whether they are mechanosensitive themselves and, if so, what is the functional significance of this mechanosensitivity. Is it needed for touch? Does the mechanoresponse of an accessory cell precede that of the nerve ending or is it simultaneous? While direct mechanosensitivity has been established for Merkel cells and is starting to emerge for the Meissner and the Pacinian corpuscles in vertebrate (Ikeda et al., 2014; Ranade et al., 2014; Woo et al., 2014; Nikolaev et al., 2020, 2023), as well as for the Amsh glia in *C. elegans* (Fernandez-Abascal et al., 2022), it is currently not clear whether this is a common feature across touch receptors. The emerging progress with genetically encoded sensors, combined with the conservation of general mechanisms across species, as observed in current studies, will help address some of these important questions.

## Author contributions

DL: Visualization, Writing – original draft, Writing – review and editing. JH: Visualization, Writing – review and editing. LB: Conceptualization, Funding acquisition, Project administration, Supervision, Validation, Visualization, Writing – original draft, Writing – review and editing.
